# *Staphylococcus aureus* Regulatory RNAs as Potential Biomarkers for Bloodstream Infections

**DOI:** 10.3201/eid2209.151801

**Published:** 2016-09

**Authors:** Valérie Bordeau, Anne Cady, Matthieu Revest, Octavie Rostan, Mohamed Sassi, Pierre Tattevin, Pierre-Yves Donnio, Brice Felden

**Affiliations:** University of Rennes 1, Rennes, France (V. Bordeau, A. Cady, M. Revest, O. Rostan, M. Sassi, P. Tattevin, P.-Y. Donnio, B. Felden);; Institut National de la Santé et de la Recherche Médicale Unité 835, Rennes (V. Bordeau, M. Sassi, P. Tattevin, B. Felden);; Rennes University Hospital, Rennes (A. Cady, M. Revest, P. Tattevin, P.-Y. Donnio)

**Keywords:** Staphylococcus aureus, bacteria, staphylococci, small RNAs, small pathogenicity island RNAs, spr RNAs, RNAIII, transfer–messenger RNA, biomarkers, bloodstream infections, nasal colonization, nasal carrier, sepsis, septic shock, immune evasion protein, multilocus sequence typing, S. aureus protein A typing, spa typing, second immunoglobulin-binding protein

## Abstract

*Staphylococcus aureus* is a commensal bacterium and pathogen. Identifying biomarkers for the transition from colonization to disease caused by this organism would be useful. Several *S. aureus* small RNAs (sRNAs) regulate virulence. We investigated presence and expression of 8 sRNAs in 83 *S. aureus* strains from 42 patients with sepsis or septic shock and 41 asymptomatic colonized carriers. Small pathogenicity island sRNAs sprB and sprC were clade specific. Six sRNAs had variable expression not correlated with clinical status. Expression of RNAIII was lower in strains from septic shock patients than in strains from colonized patients. When RNAIII was associated with expression of sprD, colonizing strains could be discriminated from strains in patients with bloodstream infections, including patients with sepsis and septic shock. Isolates associated with colonization might have sRNAs with target expression different from those of disease isolates. Monitoring expression of RNAIII and sprD could help determine severity of bloodstream infections.

## Abstract 2

These RNAs predict disease severity and provide targets for therapeutic approaches.

*Staphylococcus aureus* causes many community-acquired, healthcare–related, and nosocomial infections in humans. This bacterium is a commensal organism (part of the normal microflora), but it can also infect the body at various sites. Diseases caused by *S. aureus* differ greatly, ranging from skin lesions to invasive infections. A total of 20%–30% of the healthy population is colonized with *S. aureus* in the nostrils (*1*), and a substantial percentage of *S. aureus* bacteremia originates from endogenous colonies in the nasal mucosa (*2*,*3*).

Clinical expression of sepsis covers a continuum of manifestations; the most serious form is septic shock. In this state, vascular offense and systemic inflammation lead to endangered cardiac function and decrease in blood pressure that cause impaired oxygen delivery, organ failure, and death. Sepsis-related deaths and lack of mitigating clinical approaches attest to our limited understanding of the complex host–*S. aureus* interactions.

*S. aureus* has high rates of transmission and increased levels of antimicrobial drug resistance and produces many virulence factors (*4*). To coordinate expression of virulence genes during infection, *S. aureus* uses 2-component systems, transcription factors (*5*) and regulatory or small RNAs (sRNAs), which function as positive (*6*) or negative (*7*) virulence determinants. There are ≈160 sRNAs in the Staphylococcal Regulatory RNA database (*8*). Although their functions have not been extensively investigated, some sRNAs are known to regulate virulence factors. Quorum sensing is mediated by the accessory gene regulator *agr*, and RNAIII is the effector (*9*). Staphylococcal infection severity is based on host factors and bacterial pathogenesis (*10*). We investigated differences in sRNA gene content and expression levels in *S. aureus* strains isolated from patients with bloodstream infections and from asymptomatic carriers.

## Materials and Methods

### Isolates and Sample Collection

We obtained clinical isolates from a prospective study of all patients given a diagnosis of *S. aureus* bloodstream infections in 2006 at the Rennes University Hospital (Rennes, France), a tertiary referral hospital in western France. We selected patients with nonsevere sepsis or septic shock (*11*). Patients with severe sepsis (sepsis with organ dysfunction or tissue hypoperfusion improving after fluid therapy and not requiring vasopressors) were not included because their clinical status might too closely resemble nonsevere sepsis or shock. To prevent other confounding factors, immunodeficient patients were excluded: those infected with HIV; those with congenital immunodeficiency, malignant hemopathy, organ or stem cell transplants; those receiving systemic corticosteroid therapy for >3 weeks; and those undergoing another immunosuppressive treatment.

We extracted data from medical records. A nosocomial bloodstream infection was defined as either a bloodstream infection diagnosed in a patient hospitalized for >48 hours before symptom onset or a bloodstream infection in a patient receiving chronic hemodialysis or peritoneal dialysis. For each patient, we calculated at admission the Charlson Comorbidity Index and the Simplified Acute Physiology Score (*12*). We also collected 41 isolates from asymptomatic carriers: 23 from medical students in Rennes; 7 from healthcare workers sampled during their medical visit at a hospital in Lausanne, Switzerland; and 11 from the National Reference Laboratory for Staphylococci in Lyon, France. The study was approved by review board at Rennes University Hospital.

### Multilocus Sequence Typing and *S. aureus* Protein A Typing

*S. aureus* protein A (*spa*) typing was performed by using primers spa-1113f (5′-TAAAGACGATCCTTCGGTGAGC-3′) and spa-1514r (5′CAGCAGTAGTGCCGTTTGCTT-3′). Sequences were determined by using a BigDye Terminator v3.1 Cycle Sequencing Kit (Applied Biosystems, Foster City, CA, USA) and a 3730xl DNA analyzer (Applied Biosystems). The *spa* repeats and types were determined by using BioNumerics (Applied Maths, Sint-Matens-Latem, Belgium) and the Ridom *Spa* Server (http://www.spaserver.ridom.de/). The *spa* types with similar profiles were grouped within similar lineages. Multilocus sequence typing (MLST) was performed according to procedures of Enright et al. (*13*). The PCR products were sequenced by using a 3730xl DNA analyzer, and sequence types (STs) were determined by using BioNumerics and the MLST database (http://www.mlst.net/). MecA1 and MecA2 primers were used to amplify a 1,102-bp gene fragment, which was used to identify *mecA*. Isolates were screened for *tst* and *pvl* by using real-time PCR. PCR was used to detect small pathogenicity island (PI) RNAs (spr); sprA1/2, sprB, sprC, sprD, sprX, ssrA, 6S RNA, and rsaE. Primers used are listed in Technical Appendix Table). All PCR products were analyzed by using 2% agarose gel electrophoresis.

### Bacterial Cultures, RNA Isolation, and Expression Analysis

*S. aureus* strains were grown in Luria-Bertani medium and then harvested. Cells were isolated by centrifugation and dissolved in a solution of 33 mmol/L sodium acetate, 17 mmol/L sodium dodecyl sulfate, and 1 mmol/L EDTA (pH 5.5). The cells were then mixed with glass beads and lysed by using a Fast Prep Apparatus (MP Biochemicals, LLC, Santa Ana, CA, USA).

RNAs were isolated by using water-saturated phenol (pH 5.0). RNAs were precipitated and washed with ethanol. Northern blotting of RNA markers was conducted by loading 10 μg of total RNA onto 8 mol/L urea, 8% polyacrylamide gels. Gels were subjected to electrophoresis and blotted onto nylon membranes at 30 V for 1.5 h in 0.5× Tris-HCl, borate, EDTA buffer.

Prehybridization and hybridization were performed by using ExpressHyb solution (Clontech, Mountain View, CA, USA) and ^32^P-labeled DNAs (Technical Appendix Table 2). Signals were detected by using phosphorimaging (Biocompare, South San Francisco, CA, USA) and quantified. Expression levels of sRNAs in strains were monitored by using quantitative PCR and specific primers (Technical Appendix Table 3). cDNAs were produced by using a High-Capacity cDNA Reverse Transcription Kit (Applied Biosystems). Using the comparative cycle threshold method (Applied Biosystems), we normalized sRNA counts against transfer–messenger RNA (tmRNA) and *S. aureus* reference strain L102.

### Bacterial Protein Extracts and Western Blotting

For preparation of protein extracts, bacteria were grown until the desired optical density (OD) at 600 nm was reached. Cells were centrifuged at 8,000 × *g* for 10 min at 4°C and suspended in lysis buffer (10 mmol/L Tris-HCl [pH 7.5], 20 mmol/L NaCl, 1 mmol/L EDTA, and 5 mmol/L MgCl_2_) in the presence of a protease inhibitor cocktail tablet containing 0.1 mg/mL lysostaphin. The cells was then dissolved in 1× Laemmli buffer containing 10% β-mercaptoethanol and heated at 90°C for 5 min. Samples were separated by sodium dodecyl sulfate–polyacrylamide gel electrophoresis on 8% polyacrylamide gels and transferred to polyvinylidene fluoride membranes at 100 V for 1 h. Membranes were blocked in Tris-buffered saline containing 5% milk.

Second immunoglobulin-binding protein (Sbi), which is an immune evasion protein, was detected by using specific antibodies as described (*14*), and SaeR protein was detected by using specific antibodies. Incubation with primary antibodies against Sbi (diluted 1:10,000) or antibodies against SaeR protein (diluted 1:5,000) was performed at room temperature for 2 h. Blots were incubated with antirabbit IgG peroxidase-conjugated secondary antibodies for 1 h; washed in Tris-buffered saline, 0.05% Tween; developed in ECL Western Blotting Detection Reagent (GE Healthcare Bio-Sciences, Pittsburgh, PA, USA); and exposed on an ImageQuant LAS4000 Imaging System (GE Healthcare Bio-Sciences). Quantifications were performed by using the ImageQuant System. Levels of Sbi or SaeR protein were normalized against levels of total proteins.

All statistical tests and graphic representations were performed by using GraphPad Prism software (GraphPad Software Inc., La Jolla, CA, USA). Quantitative values were compared by using the Mann-Whitney U test. A p value <0.05 was considered significant.

## Results

### Characteristics of Patients with *S. aureus* Bloodstream Infections

A total of 42 patients (17 with septic shock and 25 with nonsevere sepsis) were included in this study (Table 1). Patients with nonsevere sepsis were more likely than those with septic shock to have nosocomial bloodstream infections (p = 0.02) and a lower Simplified Acute Physiology Score (p = 0.01). The mortality rate was also significantly higher for patients with septic shock than for patients with nonsevere sepsis (41.2% vs. 8.0%; p = 0.01). The clonal distribution of isolates (Table 2) was similar to that reported for France in the European Antimicrobial Resistance Surveillance System (*15*).

**Table 1:** Clinical characteristics of 42 patients with *Staphylococcus aureus* bloodstream infections admitted to Rennes University Hospital, Rennes, France*

Characteristic	Sepsis, n = 25	Septic shock, n = 17	p value
Male sex, %	84	84	1.00
Age, y	62.7 (15–97)	68.1 (33–84)	0.30
Nosocomial bacteremia	80	41.1	0.02
MRSA	32	5.8	0.06
Diabetes mellitus	16	29.4	0.45
Alcohol abuse	12	35.3	0.12
Charlson Comorbidity Index	1.4 (0–5)	2.1 (0–5)	0.06
Endovascular device	52	29.4	0.21
SAPS II	43.2 (14–61)	60.9 (38–126)	0.01
Delayed antibiotherapy	4.6 (0–42)	3.1 (0–10)	0.42
Infective endocarditis	12	17.6	0.70
C-reactive protein	186.4 (32–427)	250.5 (67–445)	0.17
Polynuclear neutrophils	14,588 (4,700–33,000)	14,977 (4,230–26,000)	0.84
Mortality rate	8	41.2	0.01

*Values are % or no. (range). MRSA, methicillin-resistant *S. aureus;* SAPS II, Simplified Acute Physiology Score.

**Table 2:** Characteristics of 83 clinical strains of *Staphylococcus aureus*, Rennes, France*

Strain ID	Strain clinical status	MLST sequence type	*spa* type	Small pathogenicity island RNAs					Core genome RNAs		
				sprA	sprB	sprC	sprD	sprX	tmRNA	6S RNA	RNAIII
Group 1											
L253	Carriage	10	ZZ2PNGKBKGOLB	+	–	+	–	–	+	+	+
L341	Carriage	398	XKAOAOBO	–	–	+	+	–	+	+	+
7	Carriage	398	XKAOAOBO	–	–	+	+	–	+	+	+
9†	Carriage	398	XKAOAOBO	–	–	+	+	–	+	+	+
16	Carriage	398	XKAOAOBO	–	–	+	+	–	+	+	+
18	Carriage	398	XKAOAOBO	–	–	+	+	–	+	+	+
20†	Carriage	398	XKAOAOBO	–	–	+	+	–	+	+	+
430‡	Septic shock	398	XKAOAOBO	–	–	+	+	–	+	+	+
27‡	Septic shock	30	WGKAKAOAOMQ	+	–	–	+	+	+	+	+
23‡	Carriage	30	WGKAKAOMQQ	+	–	–	+	+	+	+	+
13	Carriage	30	WGKAKAOMQQ	+	–	–	+	+	+	+	+
22	Carriage	30	WGKAKAOMQ	+	–	–	+	+	+	+	+
28	Carriage	30	XKAKAOMQQ	+	–	–	+	+	+	+	+
52†‡	Sepsis	30	WGKAKAQ	+	–	–	+	+	+	+	+
46†‡	Sepsis	30	WGKAKBMQ	+	–	–	+	+	+	+	+
82†‡	Sepsis	30	WXKAKAOMQQ	+	–	–	+	+	+	+	+
2167†‡	Carriage	30	WGKBKAOMQQ	+	–	–	+	+	+	+	+
1954†‡	Carriage	30	WGKAKAOMQQQQ	+	–	–	+	+	+	+	+
KS‡	Septic shock	30	WGKAKAOMQQ	+	–	–	+	+	+	+	+
886‡	Septic shock	30	WGKAKAOMQQ	+	–	–	+	+	+	+	+
86‡	Sepsis	34	ZZ2PNGKBKGOLB	+	–	–	+	+	+	+	+
89‡	Septic shock	34	ZZ2PNGKBKGOLB	+	–	–	+	+	+	+	+
59‡	Sepsis	34	ZZ2PNGKBKGOMLLB	+	–	–	+	+	+	+	+
45‡	Septic shock	45	XKAKBEMB	+	–	–	–	–	+	+	+
34‡	Septic shock	45	XKAKBEMBKB	–	–	–	+	+	+	+	+
8‡	Carriage	45	XKAKEEMBKB	–	–	–	+	+	+	+	+
49‡	Sepsis	45	XKAKBEMBKB	–	–	–	+	+	+	+	+
2203†‡	Carriage	45	XKAKAKBEMBBKB	–	–	–	+	+	+	+	+
1911†‡	Carriage	45	A2AKBEEMBKBB	–	–	–	+	+	+	+	+
30‡	Septic shock	22	TJEJNF2MOMOK	+	–	–	+	+	+	+	+
L317	Carriage	22	TJEJNCMOMOKR	+	–	–	+	+	+	+	+
1955†‡§	Carriage	22	TJJEJNF2MNF2MOMOKR	+	–	–	+	+	+	+	+
2752‡	Carriage	22	TJEJCMOMOKR	+	–	–	+	+	+	+	+
6	Carriage	21	I2Z2EGMJH2M	–	+	–	+	+	+	+	+
14‡¶	Septic shock	121	I2Z2EGMMJH2M	+	+	–	+	+	+	+	+
Group 2											
18†‡	Sepsis	1	UJFKBPE	+	+	+	+	+	+	+	+
5	Carriage	5	TJMBMDMGMK	+	+	+	+	+	+	+	+
21‡	Carriage	5	TJMEMDMGGK	+	+	+	+	+	+	+	+
62‡§¶	Sepsis	5	TMDGGK	+	+	+	+	+ (2)	+	+	+
21	Carriage	5	TJMBMK	+	+	+	+	+	+	+	+
1899†	Carriage	5	TJMBMK	+	+	+	+	+	+	+	+
1414†	Carriage	5	TMDMGGMK	+	+	+	+	+	+	+	+
15†‡¶	Carriage	5	TJMBMDMGMK	+	+	+	+	+	+	+	+
88†‡¶	Sepsis	5	TJMBMDMGMK	+	+	+	+	+	+	+	+
104‡	Sepsis	7	UJFMBGJAGJ	+	–	+	+	+	+	+	+
17†‡¶	Septic shock	8	YHGFMBQBLO	+	+	+	–	–	+	+	+
26†‡§¶	Septic shock	8	YHGFMBQBLO	+	+	+	+	+	+	+	+
28‡¶	Septic shock	8	YHGFMBQBLO	+	+	+	+	+	+	+	+
10†‡¶	Carriage	8	YHGFMBQBLO	+	+	+	+	+	+	+	+
L102†‡¶	Carriage	8	YHGFMBQBLO	+	+	+	+	+	+	+	+
L385	Carriage	8	YHBQBLO	+	+	+	+	+	+	+	+
L400	Carriage	8	YHFMBQBLO	+	+	+	+	+	+	+	+
1†‡§¶	Carriage	8	YHFMBQBLO	+	+	+	+	+	+	+	+
2§	Carriage	8	YHFMBQBLO	+	+	+	+	+	+	+	+
3§	Carriage	8	YHFMBQBLO	+	+	+	+	+	+	+	+
11‡	Carriage	8	YHGFMBQBLO	+	+	+	+	–	+	+	+
9†‡§¶	Sepsis	8	YHGFMBQBLO	+	+	+	+	+	+	+	+
20‡§	Sepsis	8	YC2FMBQBLO	+	+	+	+	+	+	+	+
43†‡§¶	Sepsis	8	YHGFMBQBLO	+	+	+	+	+ (2)	+	+	+
75†‡¶	Sepsis	8	YMBQBLO	+	+	+	+	+	+	+	+
100§	Sepsis	8	YHGFHGFMBQBLO	+	+	+	+	+	+	+	+
2155†‡	Carriage	8	YMBQLO	+	+	+	+	+	+	+	+
310†‡	Carriage	8	YHMBQBQBLO	+	+	+	+	+	+	+	+
54‡§	Sepsis	8	YHGFMBQBLO	+	+	+	+	+	+	+	+
58‡	Sepsis	8	YHGFMBQBLO	+	+	+	+	+	+	+	+
85‡	Sepsis	8	YHGFMBQBLO	+	+	+	+	+	+	+	+
101‡§	Sepsis	8	YHGFMBQBLO	+	+	+	+	+	+	+	+
11‡	Septic shock	15	UJGBBGGJAGJ	–	+	+	+	+	+	+	+
42‡	Septic shock	15	UJGBBGGJAG	–	+	+	+	+	+	+	+
106	Sepsis	15	UJGBBGGJ	+	+	+	+	–	+	+	+
4	Carriage	15	UJGBGGJAGJ	+	+	+	+	+	+	+	+
L45‡	Carriage	15	TJGBGGJAGJ	–	+	+	+	+	+	+	+
19†‡¶	Carriage	25	ZFGU2DMGM	+	+	+	+	+	+	+	+
91†‡¶	Sepsis	25	ZFGDMGM	+	+	+	+	+	+	+	+
61‡	Sepsis	25	ZFGU2DMGGMM	+	+	+	+	+	+	+	+
74‡	Sepsis	25	ZGU2DMGGM	+	+	+	+	+	+	+	+
71†‡	Sepsis	9	I2GJAABB	+	–	+	+	+	+	+	+
44†‡¶	Septic shock	101	ZDGMDMGMM	+	–	+	+	+ (2)	+	+	+
57‡	Septic shock	188	UJGFMB	+	+	+	+	+	+	+	+
26‡	Carriage	188	UJGFMB	+	+	+	+	+	+	+	+
12	Carriage	883	JJMMJJJJMK	+	–	+	–	–	+	+	+
69‡	Septic shock	ND	GFMGGM	+	+	+	–	–	+	+	+
65†§¶	Sepsis	ND	ND	+	+	+	+	+	+	+	+

*Values in parentheses indicate number of detected gene copies. ID, identification; MLST, multilocus sequence typing; spa, *S. aureus* protein A; spr, small pathogenicity island RNA; tm, transfer–messenger; +, positive; –, negative; ND, not determined. †Strains for which SaeR expression was analyzed by Western blot. ‡Strains for which quantitative PCR results and second immunoglobulin-binding protein expression was analyzed by Western blot. §Strains resistant to methicillin. ¶Strains for which small RNA expression was analyzed by Northern blot.

### Genotyping of Strains from Patients with Invasive Diseases and Asymptomatic Carriers

**Figure 1:**
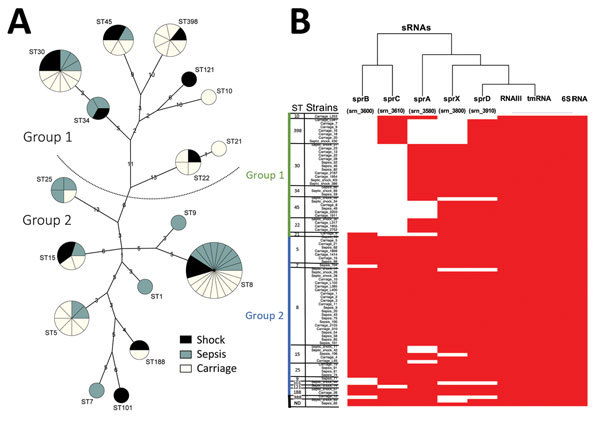
Phylogenetic and molecular typing of *Staphylococcus aureus* by using small RNAs (SRNAs), Rennes, France. A) Phylogenetic tree showing sRNA gene content and expression. Genotyping by multilocus sequence typing (MLST) and the maximum-parsimony method classified 83 *S. aureus* isolates (40 from patients with bloodstream infections and 40 from nasal carriers). Strains were distributed into 17 sequence types (STs) in groups 1 and 2 according to the method of Cooper and Feil (*16*). Three isolates were not included in the tree because 2 could not be typed and 1 belonged to the divergent ST883 lineage. Numbers indicate genetic distances between nodes. Circle sizes are proportional to number of isolates, and colors indicate origin. B) sRNA gene distribution among 83 analyzed strains. Five sRNAs were from pathogenicity islands and 3 sRNAs were from the core genome. Red indicates presence of sRNA and white indicates absence of SRNA. Heat map was constructed by using R software (https://www.r-project.org/). tmRNA, transfer–messenger RNA; ND, not determined.

We used MLST and spa typing to analyze 83 *S. aureus* isolates from 42 blood cultures for patients with bloodstream infections or 41 nasal samples from asymptomatic carriers. Isolates clustered into 17 STs (Figure 1, panel A). Of 83 strains analyzed, none had genes encoding the Panton-Valentine leukocidin, which is associated with increased virulence of certain strains. Toxic shock syndrome toxin genes were detected in infectious and methicillin-susceptible *S. aureus* colonizing strains that belonged to ST5 and ST30.

Among 37 methicillin-susceptible *S. aureus* colonizing strains, ST398 was the most common (n = 6) (Technical Appendix Table 4). Only 4 strains isolated from nasal samples were methicillin-resistant *S. aureus* (MRSA), and these belonged to ST8 and ST22 (Table 2). Most (n = 10) MRSA isolates were ST8 (Table 2). MRSA prevalence was ≈10% in healthy colonized healthcare workers and students and ≈2% in the general population (*17*). The prevalence of MRSA in infectious samples (≈21%), all positive for *mecA*, was consistent with the overall prevalence of staphylococcal infections in France (*18*). We detected a predominance of the ST8 MRSA clone, which is the predominant pandemic MRSA clone in France and has *sea* and *lukED* genes. Isolates from patients with bloodstream infections and carriers were evenly distributed among the STs (Table 2). As reported by Feil et al. (*19*), genetic distances between the 8 group 1 STs (mean ± SD 25.2 ± 10.6) were longer than those between the 9 group 2 STs (20.0 ± 7.4), which is consistent with earlier emergence of group 1 isolates.

### Specificities of sRNAs for Bacterial Clades

We used PCR to identify a subset of sRNAs that were specific for conserved sequences. Eight sRNAs were selected from core and accessory genomes for determining their distribution among strains. We chose sRNAs according to their presence in the accessory genome because this presence implies variability in their presence/absence among strains and their putative roles in virulence. We included the few sRNAs ubiquitously detected in bacteria. Housekeeping tmRNA and 6S RNA genes, which were detected in many bacterial species, were found in all strains. All strains also contained RNAIII, which is the quorum-sensing effector (*9*).

In addition, we detected 5 sRNAs expressed on PIs: sprA (srn_3580), sprB (srn_3600), sprC (srn_3610), sprD (srn_3800), and sprX (srn_3820) (*8*). Because of absence of PIs *phiSa3* and *vsaβ* in group 1, all 5 sRNAs on PIs were detected only in group 2 STs (Table 2). We found that sprA was rarely detected in ST398 strains, sprB was not detected in all group 1 strains and STs, sprC was not detected in group 1 STs except ST398 strains, sprD was detected in all but 5 strains from both groups, and sprX was detected in all strains except ST398. Detection of sprD and sprX in most STs from both groups reflects evolution of S. aureus, which has been punctuated by successive acquisitions and losses of genetic elements. The presence of sprB and sprC among *S. aureus* infectious isolates indicates *S. aureus* phylogeny and strain clonality (Figure 1, panel B). Strain genotyping showed that the sample analyzed reflected diversity of staphylococcal infections at the national level in France.

### PI-Encoded RNA Expression

**Figure 2:**
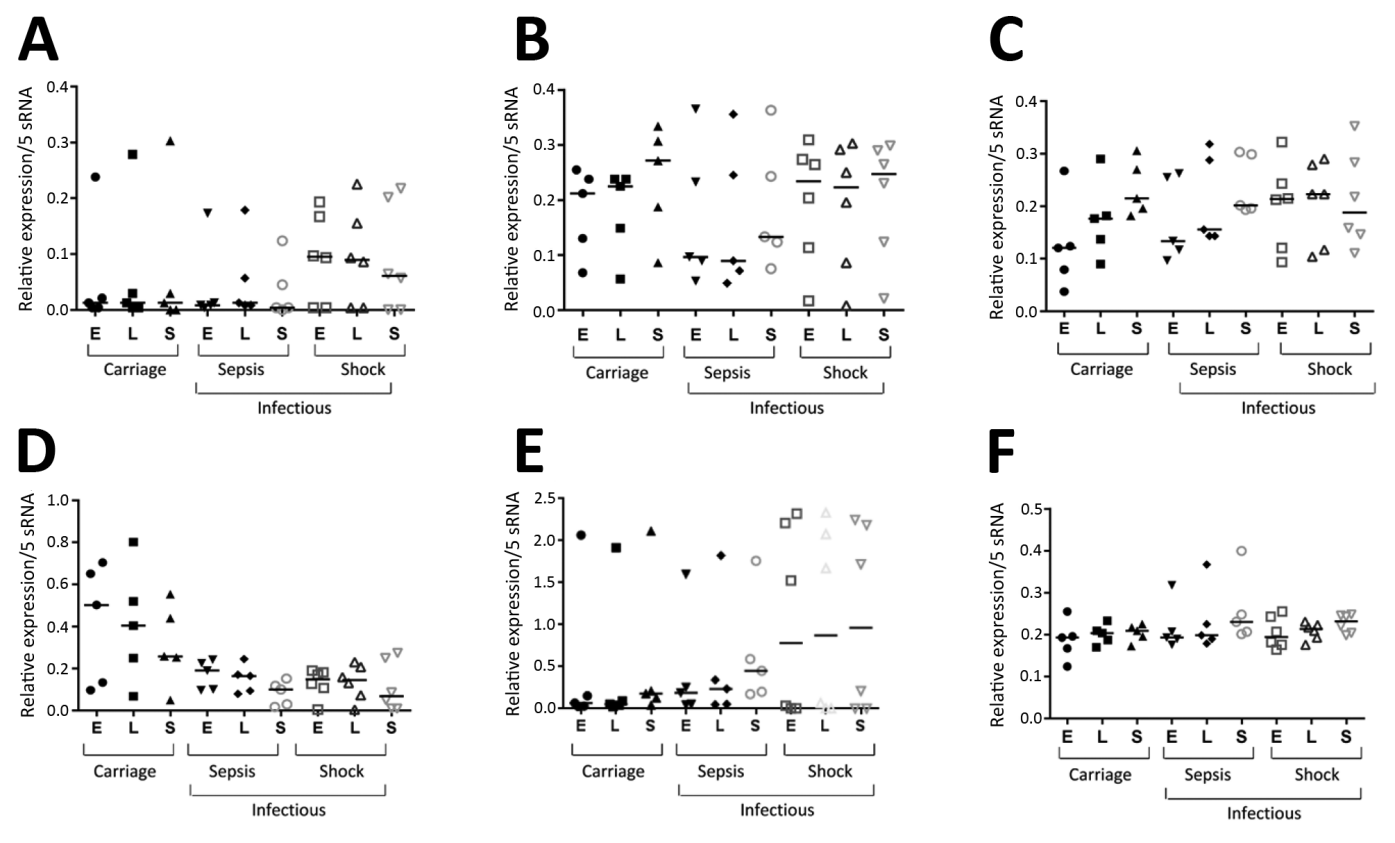
Expression of 5 sRNAs and tmRNA in 16 *Staphylococcus aureus* isolates from patients with bloodstream infections (nonsevere sepsis or septic shock) and asymptomatic colonized carriers (carriage), Rennes, France. A) sprA; B) sprB; C) sprC; D) sprD; E) sprX; F) tmRNA. Isolates were derived from ST5, ST8, and ST25. Expression was measured by using Northern blotting after strain isolation and culture. Total RNAs were obtained during early exponential (E), late exponential (L), and stationary (S) growth phases. Horizontal lines indicate medians of expression for each growth phase and sRNA. tmRNA was used as reference sRNA for subsequent quantitative PCR analyses that monitored expression levels of other sRNAs because its expression is stable for all isolates at each growth phase. Black dots indicate carriage strains at E growth phase; black squares indicate carriage strains at L growth phase; black triangles indicate carriage strains at S growth phase; inverted black triangles indicate strains causing sepsis at E growth phase; black diamonds indicate strains causing sepsis at L growth phase; circles indicate strains causing sepsis at S growth phase; squares indicate strains causing shock at E growth phase; triangles indicate strains causing shock at L growth phase; inverted triangles indicate strains causing shock at S growth phase. ST, sequence type; sRNA, small RNA; spr, small pathogenicity island RNA; tmRNA, transfer–messenger RNA.

**Figure 3:**
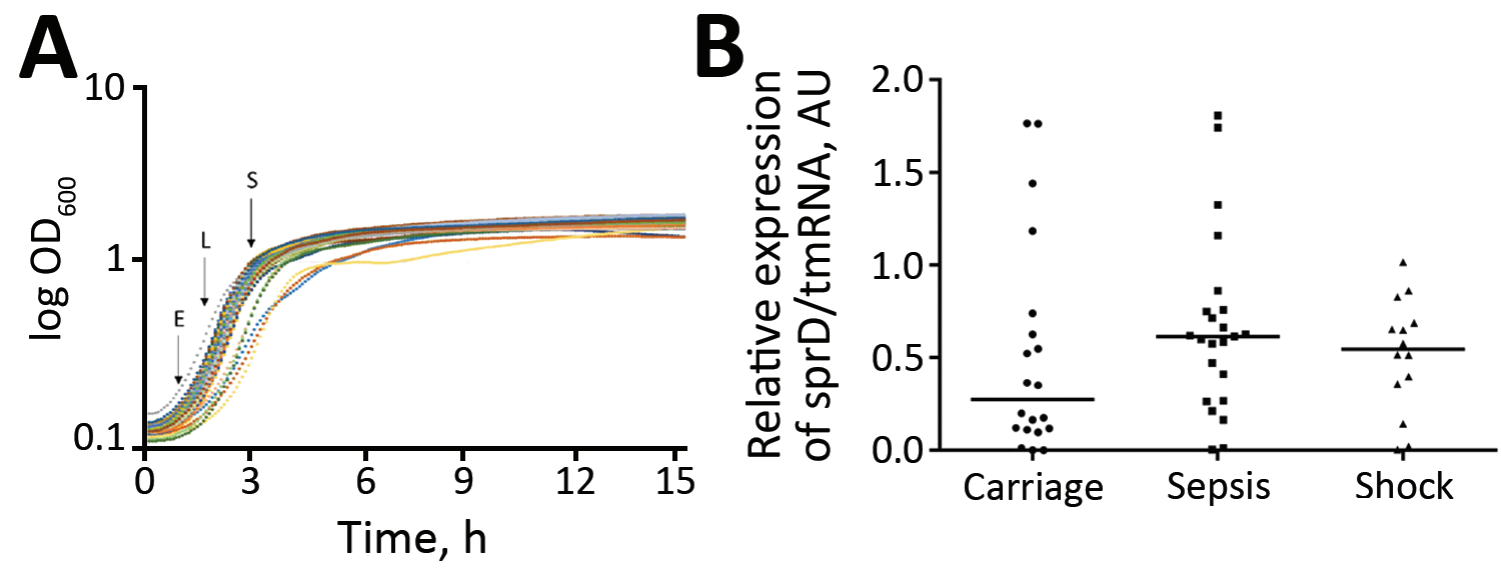
Expression of sprD in 61 *Staphylococcus aureus* isolates, Rennes, France. A) Representative growth curves. Each curve indicates 1 strain. Arrows indicate times at which the total RNAs were collected. E, early exponential growth phase; L, late exponential growth phase; S, stationary growth phase. B) Isolates analyzed for sprD (srn_3800) expression levels at E growth phase: 21 from asymptomatic carriers, 23 from patients persons with nonsevere sepsis, and 17 from patients with septic shock. For normalization, quantitative PCR was used to determine expression of tmRNA for each isolate as internal loading controls. Horizontal lines indicate medians. Using the comparative cycle threshold method, we normalized the amount of sprD against that of tmRNA relative to that of control strain L102 (methicillin-susceptible *S. aureus* colonization strain). Each symbol indicates mean for 3 independent experiments. p = 0.09 for isolates from asymptomatic carriers versus isolates from patients with sepsis. AU, arbitrary units; log OD_600_, log optical density at 600 nm; ST, sequence type; sRNA, small RNA; spr, small pathogenicity island RNA; tmRNA, transfer–messenger RNA.

Because of low amounts (10–100 CFU/mL of blood) of bacteria isolated from patients with bloodstream infections (*20*), *S. aureus* isolates must be cultured before assessing sRNA expression. We selected 16 strains for subsequent analyses: 5 from nasal carriers, 6 from patients with sepsis, and 5 from patients with septic shock. Each group of samples contained strains from the same sequence types (ST5, ST8, and ST25) (Table 2). We intentionally included strains belonging to the same ST (ST8), with strains from nasal carriers, from patients with sepsis, and from patients with septic shock. Strain selection was also dictated by availability in our collection. For these 16 isolates, we assessed sRNA expression levels at OD_600 nm_ = 2 (early exponential), OD_600 nm_ = 4 (late exponential), and OD_600 nm_ = 8 (stationary) growth phases (Figure 2; Technical Appendix Figure). Growth curves for all isolates were superimposable (Figure 3, panel A).

We compared overall sRNA expression levels among the infectious subgroups. For all strains, tmRNA was constitutively expressed, and there were no differences in expression among strains (Figure 2). This finding is consistent with the status of the tmRNA gene as a housekeeping gene involved in ribosome rescue (*21*). The 6S RNA was also constitutively expressed, and there were no differences in expression among strains. Expression of the 5 Spr RNAs varied widely among the strains (Figure 2). We found that sRNA showed different expression profiles within the same ST (Technical Appendix Figure), which indicated the complexity and variability of sRNA-driven gene regulation in *S. aureus*.

Expression of sprD was heterogeneous in strains from asymptomatic carriers but more homogeneous in infected patients (Figure 2). These results were inferred from Northern blotting performed for 3 independent RNA extractions. Afterwards, the set of analyzed strains was nearly quadrupled to 61 isolates, with 21 from carriers, 23 from nonsevere sepsis patients, and 17 from septic shock patients. Because Northern blotting showed variations in SprD expression levels between the clinical strain sets (Figure 2), we monitored SprD expression by using quantitative PCR at OD_600nm_ = 2 (Figure 3, panel A). Strains from asymptomatic carriers and sepsis patients expressed SprD heterogeneously, although SprD was expressed at low levels in all strains isolated from patients with septic shock (Figure 3, panel B).

### Discrimination of Isolates by Expression Levels of RNAIII and RNAIII/SprD

**Figure 4:**
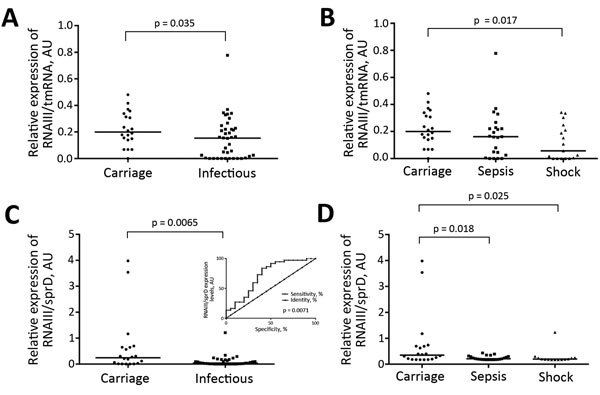
Discrimination of colonizing strains of *Staphylococcus aureus* from patients with bloodstream infections, Rennes, France. A) RNAIII analysis of strains from carriers and infected persons; B) RNAIII analysis of strains from carriers and persons with nonsevere sepsis or septic shock; C) RNAII/sprD analysis of strains from carriers and infected persons; D) RNAII/sprD analysis of strains from carriers and persons with nonsevere sepsis or septic shock. RNAIII and sprD levels were calculated relative to those for tmRNA. RNAIII expression was monitored by quantitative PCR during early exponential growth phase for 61 strains. p values (by Mann-Whitney U test) for significant differences are shown. Panel C inset shows receiver operating characteristic analysis showing discrimination of carriage strains from infectious strains. Horizontal lines indicate medians. Using the comparative cycle threshold method, we normalized the amount of RNAIII against that of tmRNA relative to that for control strain L102 (methicillin-susceptible *S. aureus* colonization strain). Each symbol indicates mean for 3 independent experiments. AU, arbitrary units; spr, small pathogenicity island RNA; tmRNA, transfer–messenger RNA.

Another RNA implicated in *S. aureus* virulence is RNAIII (*9*), which is an archetype of RNA-mediated regulation of virulence genes. Therefore, we used quantitative PCR to monitor RNAIII expression levels for 61 isolates (Figure 4) during E growth phase (Figure 3, panel A). We detected significantly lower RNAIII levels in strains isolated from patients with bloodstream infections than in those from nasal carriers (p = 0.035) (Figure 4, panel A). When we compared strains from patients with septic shock with commensal isolates, strains from patients with septic shock had significantly lower levels of RNAIII (p = 0.017) (Figure 4, panel B). Calculated RNAIII expression levels in infectious and asymptomatic persons showed a progressive decrease from carriage to nonsevere sepsis to septic shock. When we combined the effects of SprD with RNAIII, this combination substantially discriminated carriage isolates from infectious isolates (p = 0.0065) (Figure 4, panel C), carriage isolates from sepsis isolates (p = 0.018), and carriage isolates from septic shock isolates (p = 0.025) (Figure 4, panel D). We conducted receiver operating characteristic analyses to determine the capacity of differential expression of RNAIII and SprD in predicting disease outcome. Our findings indicate differences in RNAIII/SprD expression levels between colonizing strains and infectious strains (Figure 4, panel C, inset).

### Discrimination of Isolates by Expression Levels of Sbi

**Figure 5:**
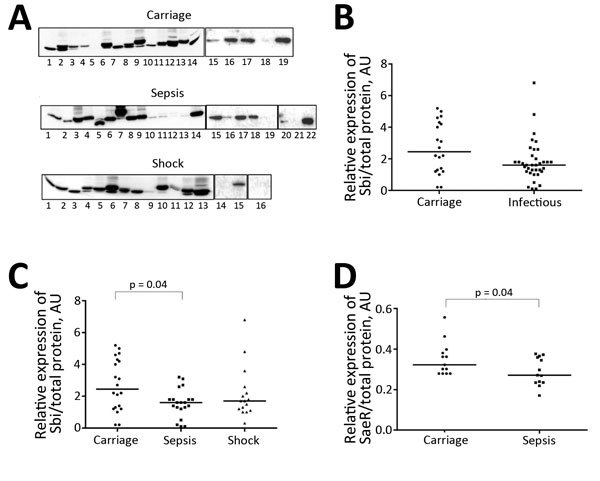
Expression levels of Sbi and SaeR proteins of *Staphylococcus aureus* isolated from patients with bloodstream infections and asymptomatic carriers, Rennes, France. A) Western blot of Sbi protein for 61 strains during early exponential growth phase. A protein sample from strain 19 was loaded on each gel and used as an internal control to prevent intensity variations of the bands between each experiment. Carriage: lane 1, 1; lane 2, 10; lane 3, 15; lane 4, 19; lane 5, L102; lane 6, 1911; lane 7, 0026; lane 8, 0310; lane 9; 2203; lane 10, 2155; lane 11, 2752; lane 12, 2167; lane 13, 1954; lane 14, 1955; lane 15, 21; lane 16, 11; lane 17, L45; lane 18, 8; lane 19, 23. Sepsis: lane 1, 49; lane 2, 18; lane 3, 88; lane 4, 82; lane 5, 46; lane 6, 71; lane 7, 59; lane 8, 75; lane 9; 91; lane 10, 86; lane 11, 9; lane 12, 43; lane 13, 20; lane 14, 52; lane 15, 85; lane 16, 104; lane 17, 54; lane 18, 58; lane 19, 61; lane 20, 101; lane 21, 62; lane 22, 74. Shock: lane 1, 26; lane 2, 44; lane 3, 17; lane 4, 14; lane 5, 42; lane 6, 30; lane 7, 34; lane 8, 45; lane 9, 57; lane 10, 89; lane 11, 27; lane 12, 11; lane 13, 28; lane 14, 430; lane 15, 069; lane 16, 886. B) and C) Sbi/total protein. D) SaeR/total protein. SaeR is a positive regulator of Sbi protein. p values (by Mann-Whitney U test) for significant differences are shown. Experiments were performed in triplicate. Horizontal lines indicate medians. To exclude loading variations between samples, values were normalized against total protein levels. AU, arbitrary units; Sbi, second immunoglobulin-binding.

SprD and RNAIII negatively regulate expression of Sbi by blocking translation through binding with the Sbi mRNA (*6**,**14*). We performed Western blotting for 61 isolates in growth phase E by using polyclonal antibodies against intracellular and membrane proteins to monitor Sbi levels (Figure 5, panel A). As reported by Smith et al. (*22*), we found that molecular weights of Sbi detected in isolates were variable (mean ≈50 kDa) and the amount of Sbi varied among isolates (Figure 5, panel A). Individual assessments showed significantly lower Sbi levels in isolates from patients with bloodstream infections than in patients with nasal colonization (p = 0.04).

## Discussion

A set of 83 *S. aureus* strains with known genotypes was collected from asymptomatic carriers and patients with nonsevere sepsis or septic shock. We used this collection for a prospective study of the presence and expression of certain sRNAs in the core and accessory genome. We also monitored expression of Sbi, an immune evasion protein whose expression is negatively controlled by the sRNAs SprD and RNAIII (*14*) and SaeR, a positive regulator of Sbi. In clinical and carriage staphylococcal strains, the presence of >2 PI-encoded sRNAs, sprB (srn_3600) and sprC (srn_3610), was indicative of the presence of PIs and prophages. These PI-encoded sRNAs, particularly sprB, could be used as probes to improve genotyping studies. These sRNAs probably appear during the transition between ST22 and ST25 (Figure 1, panel A). The sRNA sprB is absent in most group 1 isolates. In some strains, we cannot rule out that sequence variations among the sRNA genes might hamper their amplification.

Molecular typing can identify genetic diversity of strains, which is required for epidemiologic surveillance of infections. Bacterial strain typing methods include generation of DNA banding patterns, sequencing, and hybridization. Bacterial genotyping has benefited from identification of novel locus-specific typing markers. The sRNAs might be useful probes for genotyping bacteria because their overall content varies considerably, even among closely-related strains. Furthermore, because several sRNAs are encoded by mobile genetic elements, these sRNAs reflect the acquisition/loss of virulence factors encoded by these elements and molecules that confer antimicrobial drug resistance. In phylogenetic studies, selected sRNAs located in accessory genomes might shed light on genomic diversity.

The immunologic ability of patients to eradicate pathogens is a major determinant of infection outcome, and patients with septic shock are often immunocompromised (*23*). Our data suggests that, for staphylococcal bloodstream infections, one must also consider attributes of infecting strains, including at least an immune evasion molecule and sRNAs RNAIII and sprD. The effector of the *agr* quorum-sensing system was expressed at lower levels in strains isolated from patients with bloodstream infections, especially those with septic shock, than in asymptomatic carriers. Therefore, low RNAIII levels might identify *S. aureus* isolates that are responsible for bloodstream infections, even after isolation and culture. *agr*-defective *S. aureus* is a frequent cause of bloodstream infections (*24*). Our observations concur with the previous identification of inactivating mutations in the *S. aureus agr* virulence regulator, which have been associated with poor outcomes in for patients with bloodstream infections (*3*). Coupling the expression levels of RNAIII and SprD could discriminate colonization from infection and also provide useful information about severity of bloodstream infections. However, this possibility must be confirmed with a larger set of clinical isolates.

Because sprD and RNAIII negatively control the expression of the Sbi immune evasion protein (*17*), we also monitored Sbi expression in the isolates. Sbi is an immune evasion factor (*25*) involved in the *S. aureus-*induced inflammatory response (*26*). Cell wall−anchored Sbi proteins are essential components for *S. aureus* survival in the commensal state (*27*). Detection of additional Sbi proteins in strains isolated from asymptomatic carriers than in those from patients with septic shock is consistent with their role during colonization and in immune tolerance. Expression of Sbi at the transcriptional level is positively regulated by SaeRS, which is composed of the histidine kinase SaeS and the response regulator SaeR (*28*). Expression of Sbi is controlled by >3 regulators: negatively by 2 sRNAs, and positively by a 2-component system. This system indicates why RNAIII and sprD levels are not inversely correlated with Sbi levels in isolates tested. Carriage strains have higher SaeR levels than the sepsis strains, which is consistent with higher Sbi levels in the carriage strains than sepsis strains (Figure 5).

The transition from commensalism to infection in *S. aureus* is an essential but complex process. From a clinical standpoint, most *S. aureus* infections are derived from previous colonizers (*29*). When those strains switch to invasiveness, the transition may be related to regulatory network expression changes, including within sRNAs. Sequencing has identified changes in regulatory functions of strains isolated from a person who initially was a carrier and then showed development of a fatal bloodstream infection (*30*), which suggested that molecular evolution might play a key role in this process. Our results suggest that certain sRNAs from the gene regulatory network in a human pathogen will provide insights into commensal-to-pathogen transitions. These sRNAs could be used as surrogate markers for severity of staphylococcal infections and as biomarkers for prophylaxis and monitoring of *S. aureus* infection. Comparison of frequency and expression of selected sRNAs in *S. aureus* isolates that colonize or become infectious might be a way to identify associations between sRNA expression and disease patterns.

In vitro expression levels of some *S. aureus* sRNAs might not reflect their in vivo levels (*31*). Nevertheless, direct analysis of expression levels of bacterial sRNAs directly in blood from patients with bloodstream infections is technically difficult because of low levels of bacteria collected. We compared sRNA expression in fresh isolates from patients with the same isolates after they were thawed after be frozen for 3 weeks. We found no differences in RNAIII and sprD expression between isolates obtained directly from patients and isolates that had been frozen. Subsequent investigations will address the functional and clinical relevance of expression patterns of RNAIII and sprD. Broadening our investigations to include additional sRNAs might identify biomarkers that predict staphylococcal disease severity in infected patients. In addition to their roles as biomarkers, sRNAs could also be targets for innovative therapeutic approaches.

Technical Appendix Analysis of *Staphylococcus aureus* regulatory RNAs as potential markers for bloodstream infections.

## References

[1] van Belkum A, Verkaik NJ, de Vogel CP, Boelens HA, Verveer J, Nouwen JL, Reclassification of *Staphylococcus aureus* nasal carriage types. J Infect Dis. 2009;199:1820–6 . 10.1086/599119 19419332

[2] von Eiff C, Becker K, Machka K, Stammer H, Peters G. Nasal carriage as a source of *Staphylococcus aureus* bacteremia. Study Group. N Engl J Med. 2001;344:11–6 . 10.1056/NEJM200101043440102 11136954

[3] Smyth DS, Kafer JM, Wasserman GA, Velickovic L, Mathema B, Holzman RS, Nasal carriage as a source of *agr*-defective *Staphylococcus aureus* bacteremia. J Infect Dis. 2012;206:1168–77 . 10.1093/infdis/jis483 22859823 PMC3448967

[4] Tong SY, Davis JS, Eichenberger E, Holland TL, Fowler VG Jr. *Staphylococcus aureus* infections: epidemiology, pathophysiology, clinical manifestations, and management. Clin Microbiol Rev. 2015;28:603–61 . 10.1128/CMR.00134-14 26016486 PMC4451395

[5] Rosenstein R, Gotz F. What distinguishes highly pathogenic staphylococci from medium- and non-pathogenic? Curr Top Microbiol Immunol. 2013;358:33–89 . 10.1007/978-3-662-45793-1_286 23224647

[6] Chabelskaya S, Gaillot O, Felden B. A *Staphylococcus aureus* small RNA is required for bacterial virulence and regulates the expression of an immune-evasion molecule. PLoS Pathog. 2010;6:e1000927 . 10.1371/journal.ppat.1000927 20532214 PMC2880579

[7] Romilly C, Lays C, Tomasini A, Caldelari I, Benito Y, Hammann P, A non-coding RNA promotes bacterial persistence and decreases virulence by regulating a regulator in *Staphylococcus aureus.* PLoS Pathog. 2014;10:e1003979 . 10.1371/journal.ppat.1003979 24651379 PMC3961350

[8] Sassi M, Augagneur Y, Mauro T, Ivain L, Chabelskaya S, Hallier M, SRD: a *Staphylococcus* regulatory RNA database. RNA. 2015;21:1005–17 . 10.1261/rna.049346.114 25805861 PMC4408781

[9] Novick RP, Geisinger E. Quorum sensing in staphylococci. Annu Rev Genet. 2008;42:541–64 . 10.1146/annurev.genet.42.110807.091640 18713030

[10] Powers ME, Bubeck Wardenburg J. Igniting the fire: *Staphylococcus aureus* virulence factors in the pathogenesis of sepsis. PLoS Pathog. 2014;10:e1003871 . 10.1371/journal.ppat.1003871 24550724 PMC3923759

[11] Dellinger RP, Levy MM, Rhodes A, Annane D, Gerlach H, Opal SM, Surviving sepsis campaign: international guidelines for management of severe sepsis and septic shock, 2012. Intensive Care Med. 2013;39:165–228 . 10.1007/s00134-012-2769-8 23361625 PMC7095153

[12] Christensen S, Johansen MB, Christiansen CF, Jensen R, Lemeshow S. Comparison of Charlson Comorbidity Index with SAPS and APACHE scores for prediction of mortality following intensive care. Clin Epidemiol. 2011;3:203–11 . 10.2147/CLEP.S20247 21750629 PMC3130905

[13] Enright MC, Day NP, Davies CE, Peacock SJ, Spratt BG. Multilocus sequence typing for characterization of methicillin-resistant and methicillin-susceptible clones of *Staphylococcus aureus.* J Clin Microbiol. 2000;38:1008–15. 10698988 10.1128/jcm.38.3.1008-1015.2000 PMC86325

[14] Chabelskaya S, Bordeau V, Felden B. Dual RNA regulatory control of a *Staphylococcus aureus* virulence factor. Nucleic Acids Res. 2014;42:4847–58 . 10.1093/nar/gku119 24510101 PMC4005698

[15] Dauwalder O, Lina G, Durand G, Bes M, Meugnier H, Jarlier V, Epidemiology of invasive methicillin-resistant *Staphylococcus aureus* clones collected in France in 2006 and 2007. J Clin Microbiol. 2008;46:3454–8 . 10.1128/JCM.01050-08 18667599 PMC2566079

[16] Cooper JE, Feil EJ. The phylogeny of *Staphylococcus aureus*: which genes make the best intra-species markers? Microbiology. 2006;152:1297–305 . 10.1099/mic.0.28620-0 16622047

[17] Robert J, Tristan A, Cavalie L, Decousser JW, Bes M, Etienne J, Panton-Valentine leukocidin–positive and toxic shock syndrome toxin 1–positive methicillin-resistant *Staphylococcus aureus*: a French multicenter prospective study in 2008. Antimicrob Agents Chemother. 2011;55:1734–9 . 10.1128/AAC.01221-10 21220529 PMC3067161

[18] Le Moing V, Alla F, Doco-Lecompte T, Delahaye F, Piroth L, Chirouze C, *Staphylococcus aureus* bloodstream infection and endocarditis: a prospective cohort study. PLoS One. 2015;10:e0127385 . 10.1371/journal.pone.0127385 26020939 PMC4447452

[19] Feil EJ, Cooper JE, Grundmann H, Robinson DA, Enright MC, Berendt T, How clonal is *Staphylococcus aureus?* J Bacteriol. 2003;185:3307–16 . 10.1128/JB.185.11.3307-3316.2003 12754228 PMC155367

[20] Kiehn TE, Wong B, Edwards FF, Armstrong D. Comparative recovery of bacteria and yeasts from lysis-centrifugation and a conventional blood culture system. J Clin Microbiol. 1983;18:300–4. 6352732 10.1128/jcm.18.2.300-304.1983 PMC270795

[21] Keiler KC. Mechanisms of ribosome rescue in bacteria. Nat Rev Microbiol. 2015;13:285–97 . 10.1038/nrmicro3438 25874843

[22] Smith EJ, Visai L, Kerrigan SW, Speziale P, Foster TJ. The Sbi protein is a multifunctional immune evasion factor of *Staphylococcus aureus.* Infect Immun. 2011;79:3801–9 . 10.1128/IAI.05075-11 21708997 PMC3165492

[23] Hotchkiss RS, Monneret G, Payen D. Immunosuppression in sepsis: a novel understanding of the disorder and a new therapeutic approach. Lancet Infect Dis. 2013;13:260–8 . 10.1016/S1473-3099(13)70001-X 23427891 PMC3798159

[24] Painter KL, Krishna A, Wigneshweraraj S, Edwards AM. What role does the quorum-sensing accessory gene regulator system play during *Staphylococcus aureus* bacteremia? Trends Microbiol. 2014;22:676–85 . 10.1016/j.tim.2014.09.002 25300477

[25] Burman JD, Leung E, Atkins KL, O’Seaghdha MN, Lango L, Bernado P, Interaction of human complement with Sbi, a staphylococcal immunoglobulin-binding protein: indications of a novel mechanism of complement evasion by *Staphylococcus aureus.* J Biol Chem. 2008;283:17579–93 . 10.1074/jbc.M800265200 18434316 PMC2649420

[26] Gonzalez CD, Ledo C, Giai C, Garofalo A, Gomez MI. The Sbi protein contributes to *Staphylococcus aureus* inflammatory response during systemic infection. PLoS One. 2015;10:e0131879 . 10.1371/journal.pone.0131879 26126119 PMC4488394

[27] Foster TJ, Geoghegan JA, Ganesh VK, Hook M. Adhesion, invasion and evasion: the many functions of the surface proteins of *Staphylococcus aureus.* Nat Rev Microbiol. 2014;12:49–62 . 10.1038/nrmicro3161 24336184 PMC5708296

[28] Rogasch K, Ruhmling V, Pane-Farre J, Hoper D, Weinberg C, Fuchs S, Influence of the two-component system SaeRS on global gene expression in two different *Staphylococcus aureus* strains. J Bacteriol. 2006;188:7742–58 . 10.1128/JB.00555-06 17079681 PMC1636327

[29] Wertheim HF, Melles DC, Vos MC, van Leeuwen W, van Belkum A, Verbrugh HA, The role of nasal carriage in *Staphylococcus aureus* infections. Lancet Infect Dis. 2005;5:751–62 . 10.1016/S1473-3099(05)70295-4 16310147

[30] Young BC, Golubchik T, Batty EM, Fung R, Larner-Svensson H, Votintseva AA, Evolutionary dynamics of *Staphylococcus aureus* during progression from carriage to disease. Proc Natl Acad Sci U S A. 2012;109:4550–5 . 10.1073/pnas.1113219109 22393007 PMC3311376

[31] Song J, Lays C, Vandenesch F, Benito Y, Bes M, Chu Y, The expression of small regulatory RNAs in clinical samples reflects the different life styles of *Staphylococcus aureus* in colonization vs. infection. PLoS One. 2012;7:e37294 . 10.1371/journal.pone.0037294 22629378 PMC3358344

