# Supplementary material for: Staphylococcus aureus Regulatory RNAs as Potential Biomarkers for Bloodstream Infections
Source: Emerg Infect Dis. 2016 Sep;22(9):1570–8. doi: 10.3201/eid2209.151801 (PMC4994353; doi:10.3201/eid2209.151801)
Supplement: Technical Appendix — Analysis of Staphylococcus aureus regulatory RNAs as potential markers for bloodstream infections. [file 15-1801-Techapp-s1.pdf]

Article DOI: http://dx.doi.org/10.3201/eid2209.151801

Staphylococcus aureus Regulatory RNAs as Potential Biomarkers for Bloodstream Infections

Technical Appendix

Technical Appendix Table 1. DNA primers and probes used to detect genes in 85 strains of *Staphylococcus aureus*, Rennes, France*

Gene	Primer or probe	Sequence, 5′→3′
lukED	lukED-fw	GACTTTATTCCCTAGAACTGGTATTTACG
	lukED-rev	CACTTTAATTTCATGTGTTTTCCAGTTAAC
	lukED(VIC/MGB)	AGAAAGAAAGCATAATGCA
pvl	pvl-fw	TTCACTTGTATCTCCTGAGCCTTTT
	pvl-rev	AGTACACAGTGGTTTCAATCCTTCAT
	pvl(FAM/MGB)	CATGAGAAACAGTTGCAATA
sea	sea-fw	GGAAACGGTTAAAACGAATAAGAAAA
	sea-rev	CCTGTAAATAACGTCTTGCTTGAAGA
	sea(FAM/MGB)	TGTAACTGTTCAGGAGTTG
tst	tst-fw	GCTTGCGACAATCGCTACAG
	tst-rev	GATGCTTTTGCAGTTTTGATTATTTG
	tst(VIC/MGB)	TTTTACCCCTGTTCCC
sprA1/A2	sprA-fw	GCCTATCTCTCAGGCGTCAA
	sprA-rev	GGTGCGGCTATATGTCACCT
sprB	sprB-fw	CGAACATGTCGTCAAAGCAA
	sprB-rev	CCATTCCGCATTAACTTTGG
sprC	sprC-fw	GTCAACGACCATGCGTGGA
	sprC-rev	CCATGATTTCGAAGTCTTCATA
sprD	sprD-fw	GGGCGTTTTCAAGGAGCGCC
	sprD-rev	GCTTACCTACTTCCCATAAACAAT
sprX (rsaOR)	sprX-fw	CACCCAAGCATGTCACTGGG
	sprX-rev	GGCCACTTAACAGGCTATATAG
rsaE	igr6-fw	TTGTATTCGCTTACATTATGAACAGAT
	igr6-rev	ACAAAGGGGATGGGAGAAAT
ssrA	ssrA-fw	GGGGACGTTCATGGATTCGAC
	ssrA-rev	TGGAGACGGCGGGATTTGAAC
6S	6S-fw	GGGCCAACACTTTGATCAAGGG
	6S-rev	AGTCCGTATTGCCGTAGTTATAATAGC
mecA	mecA1	GACCGAAACAATGTGGAATTGGCC
	mecA2	CACCTTGTCCGTAACCTGAATCAGC
rnaIII	RNAIIIfor	CTAGATCACAGAGATGT
	RNAIIIrev	AAAAGGCCGCGAGCTTGGGA

*fw, forward; rev, reverse; spr, small pathogenicity island RNA.

Technical Appendix Table 2. DNA probe sequences used for Northern blot analysis of expression levels of 7 small RNAs of *Staphylococcus aureus*, Rennes, France*

Small RNAs	Sequence, 5′→3′
SprA	ATGACTGGTGCTATGATGTG
SprB	GCCAGCGTTTGGCTTGCTTT
SprC	CGGCTACTACATTCGCATGT
SprD	ACCCTAGTGAGCCCGTTAAA
SprX	TACGGGAATGCTAAAGTCAT
tmRNA	CTTCGCGAAATGAAGTGGTG
5S rRNA	CGTAAGTTCGACTACCATCG

*Spr, small pathogenicity island RNA; tm, transfer–messenger.

Technical Appendix Table 3. DNA sequences of probes for quantitative PCR analysis of tmRNA, SprD, and RNAIII gene expression in *Staphylococcus aureus*, Rennes, France*

Primer	Sequence, 5′→3′
tmRNA PCR-Q AS	TCAAACGGCAGTGTTTAGCA
tmRNA PCR-S S	CACTCTGCATCGCCTAACAG
SprD PCR-Q AS	TATTGCTCCTTTTCGGGCTA
SprD PCR-Q S	ATTGATTTGGAAAGCGCAAA
RNAIII PCR-Q AS	GAAGGAGTGATTTCAATGGCACAAGATAT
RNAIII PCR-Q S	GAATTTTGTTCACTGTGTCGATAATCCATTT

*tm, transfer–messenger; Spr, small pathogenicity RNA.

Technical Appendix Table 4. Comparison of colonization and bloodstream infection isolates of *Staphylococcus aureus*, by clonal complexes, methicillin resistance, and toxic shock syndrome toxin production, Rennes, France*

Characteristic	Colonization	BSI	p value
No. isolates	41	42	
Sequence type			0.27
ST1	0	1	
ST5	6	2	
ST7	0	1	
ST8	10	12	
ST9	0	1	
ST10	1	0	
ST15	2	3	
ST21	1	0	
ST22	3	1	
ST25	1	3	
ST30	6	5	
ST34	0	3	
ST45	3	4	
ST101	0	1	
ST121	0	1	
ST188	1	1	
ST398	6	1	
ST883	1	0	
Not typeable		2	
Methicillin resistance		0.25	
MRSA	4	9	
MSSA	37	33	
TSST-1 production	0	0	0.63
Positive	4	3	
Negative	37	39	

*BSI, bloodstream infection; ST, sequence type; MRSA, methicillin-resistant *S. aureus*; MSSA, methicillin-sensitive *S. aureus*; TSST, toxic shock syndrome toxin.

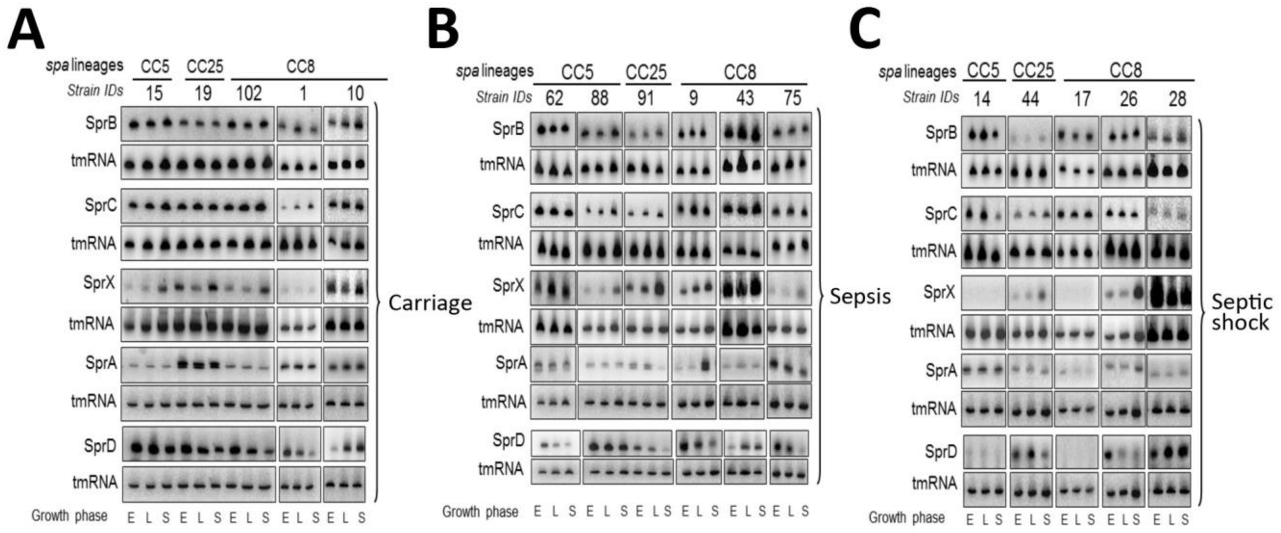

Technical Appendix Figure. Expression levels of 6 *Staphylococcus aureus* small RNAs in isolates from persons with bloodstream infections and asymptomatic colonization, Rennes, France. Isolates were detected by Northern blotting after culture. A) Carriage, B) sepsis, and C) septic shock. Isolates were derived from sequence types (STs) ST5, ST8, and ST25. Transfer–messenger RNA (tmRNA) expression was recorded for each blot for normalization against internal loading controls. *spa*, *S. aureus* protein A; CC, clonal complex; IDs, identifications; E, exponential; L, late exponential; S, stationary.
